# Axonal Protection by Netarsudil, a ROCK Inhibitor, Is Linked to an AMPK-Autophagy Pathway in TNF-Induced Optic Nerve Degeneration

**DOI:** 10.1167/iovs.63.1.4

**Published:** 2022-01-04

**Authors:** Yasushi Kitaoka, Kana Sase, Chihiro Tsukahara, Naoki Fujita, Ibuki Arizono, Jiro Kogo, Naoto Tokuda, Hitoshi Takagi

**Affiliations:** 1 Department of Molecular Neuroscience, St. Marianna University Graduate School of Medicine, Kanagawa, Japan; 2 Department of Ophthalmology, St. Marianna University School of Medicine, Kanagawa, Japan

**Keywords:** netarsudil, AMPK, autophagy, LC3, p62

## Abstract

**Purpose:**

Netarsudil, a Rho kinase inhibitor with norepinephrine transport inhibitory effect, lowers intraocular pressure, however, its effect on axon damage remains to be elucidated. The aim of the current study was to investigate the effect of netarsudil on TNF-induced axon loss and to examine whether it affects phosphorylated-AMP-activated kinase (p-AMPK) and autophagy in the optic nerve.

**Methods:**

Intravitreal administration of TNF or TNF with netarsudil was carried out on rats and quantification of axon number was determined. Electron microscopy determined autophagosome numbers. Localization of p-AMPK expression was examined by immunohistochemistry. The changes in p62, LC3-II, and p-AMPK levels were estimated in the optic nerve by immunoblot analysis. The effect of an AMPK activator A769662 or an AMPK inhibitor dorsomorphin on axon number was evaluated.

**Results:**

Morphometric analysis revealed apparent protection by netarsudil against TNF-induced axon degeneration. Netarsudil increased autophagosome numbers inside axons. Netarsudil treatment significantly upregulated optic nerve LC3-II levels in both the TNF-treated eyes and the control eyes. Increased p62 protein level induced by TNF was significantly ameliorated by netarsudil. The netarsudil administration alone lessened p62 levels. Netarsudil significantly upregulated the optic nerve p-AMPK levels. A769662 exhibited obvious axonal protection against TNF-induced damage. A769662 treatment upregulated LC3-II levels and the increment of p62 level induced by TNF was significantly ameliorated by A769662. Immunohistochemical analysis revealed that p-AMPK is present in axons. Netarsudil-mediated axonal protection was significantly suppressed by dorsomorphin administration.

**Conclusions:**

Netarsudil upregulated p-AMPK and autophagy. Netarsudil-mediated axonal protection may be associated with upregulated p-AMPK.

The Rho kinase (ROCK) inhibitors have been introduced as intraocular pressure (IOP)-lowering drugs, which improve the outflow of aqueous humor via the trabecular meshwork and Schlemm's canal.^1^ Netarsudil is a small molecule protein kinase inhibitor targeting ROCK and was approved by the US Food and Drug Administration (FDA) as eye drops in 2018.^2^ Its pharmacological effect includes dilation of episcleral veins, which lowers episcleral venous pressure.^3^ Thus, many studies have focused on its IOP lowering effect, however, its effect on optic nerve axons remains to be elucidated.

Autophagy research has received increasing attention and may need to be updated frequently. A very recent guideline has suggested that among the autophagy markers, microtubule-associated protein light chain 3 (LC3) has been used as a typically characterized autophagosome marker per se in many published studies, but the LC3 subfamily may be dispensable for autophagy regulation in certain cell types.^4^ Thus, although some assays are superseded, there are still useful assays to assess the autophagy. Basically, LC3 is initially synthesized into the cytosolic form LC3-I and then modified into the PE-conjugated form LC3-II,^5^ and this is the protein marker that is linked to autophagosomes and phagophores. Importantly, autophagy is a dynamic process and the combination of LC3-II and other protein markers, such as p62/SQSTM1, is informative for assessment of the autophagy, because it was demonstrated that inhibition of autophagy can correlate with increments of p62 levels in mammals and lessened p62 levels are correlated with autophagy induction.^4^

Adenosine monophosphate-activated protein kinase (AMPK) is a multimeric serine/threonine protein kinase, and its activity is dependent on phosphorylation on Thr172. The role of AMPK in autophagy may differ dependent on physiological or pathological condition and cell or tissue type. For example, it was shown that activation of AMPK inhibits autophagy stimulation by amino acid starvation in HEK293 cells.^6^ Conversely, it was shown that activation of AMPK phosphorylates Cyclin Y allowing the interaction of Cyclin Y with CDK16, which is needed for autophagy induction in Hela cells.^7^ In neuronal cells, a previous study showed that an AMPK activator, AICAR, promoted robust neurite outgrowth in Neuro2a cells^8^ and a recent study showed that an AMPK activator, A769662, promoted α-synuclein inclusion clearance associated with a significant reduction in p62 in SH-SY5Y cells.^9^ We and others have demonstrated beneficial roles of autophagy in optic nerve degeneration^10^^,^^11^ and retinal ganglion cell (RGC) death in the glaucoma models,^12^^–^^15^ although it is still controversial.^16^ On the other hand, a recent study showed a favorable role of the AMPK activator AICAR in protecting photoreceptors against light exposure damage,^17^ however, the role of AMPK in optic nerve axonal survival after injury remains to be clarified. The purpose of the present study is to investigate the effect of netarsudil on tumor necrosis factor (TNF)-induced optic nerve damage and to examine whether it affects autophagy. The TNF intravitreal injection model showed that axon loss preceded RGC death and recently has been used for identifying the molecular mechanism in axon degeneration.^18^ We also examined the changes in phosphorylated-APMK (p-AMPK) after netarsudil treatment, the effect of AMPK activator in TNF-induced axonal loss, and p-AMPK localization in the retina and the optic nerve.

## Materials and Methods

### Animals

Eight-week-old male Wistar rats were fed on a standard diet and water ad libitum and housed in cages (23 ± 1°C; humidity at 55 ± 5%; light from 6 AM to 6 PM). All experiments were approved by the Ethics Committee of the Institute of Experimental Animals of St. Marianna University School of Medicine and conducted according to the ARVO Statement for the Use of Animals in Ophthalmic and Vision Research.

### Intravitreal Administrations

Under anesthetization with intramuscular injections of a mixture of ketamine-xylazine, 10 ng TNF (Sigma-Aldrich, St. Louis, MO, USA) in 2 µL of phosphate-buffered saline (PBS) was administered intravitreally into the left eyes of the rats.^19^ PBS was administered intravitreally into the contralateral right eye as a control. Netarsudil mesylate (Aerie Pharmaceuticals, Inc., Durham, NC, USA) was diluted in PBS and concurrent injection of 2, 20, or 200 pmol netarsudil and TNF was carried out intravitreally. The netarsudil alone administration group was also evaluated. A769662 (an AMPK activator, Abcam, Cambridge, UK) was diluted in dimethyl sulfoxide (DMSO) and simultaneous injection of 200 pmol A769662 and TNF was carried out intravitreally. The A769662 alone administration group was also evaluated. For this experiment, DMSO was used as a control. Dorsomorphin (an AMPK inhibitor; Abcam) was diluted in DMSO and pre-injected intravitreally 1 hour before second injection of 200 pmol netarsudil plus TNF. DMSO was also pre-injected intravitreally 1 hour before the second injection of DMSO, TNF, or 200 pmol netarsudil plus TNF.

### Quantification of Axon Number

In this experiment, we used 30, 12, and 20 rats for the netarsudil study, the A769662 study, and the dorsomorphin study, respectively. Optic nerves were isolated 2 weeks after intravitreal administration, fixed, processed, and embedded in acryl resin.^19^ Semi-thin sections were made and stained 1% paraphenylene-diamine (PPD) in methanol. Center and quadrant areas (approximately 140 µm from the central) were captured with a microscopic camera and these five different images (each area is 5850 µm^2^, and total area is 29,250 µm^2^ per eye) were used to quantify the axon number for each sample using an image processing software (Aphelion, ADCIS S.A., Hérouville Saint-Clair, France).

### Electron Microscopy

Ultrathin sections (100 nm) were made and incubated with saturated uranyl acetate. Ultrastructure of optic nerves was evaluated by a transmission electron microscope (JEM-1400Plus; JEOL, Tokyo, Japan). Autophagosome numbers inside axons were determined as the sum in 10 different areas of 33.64 µm^2^ each totaling 336.4 µm^2^ per optic nerve from each sample. The analysis was performed in three to four eyes per experimental condition.

### Immunoblot Analysis

In this experiment, we used 48 and 36 rats for the netarsudil study and for the A769662 study, respectively. Optic nerves were isolated 1 week after intravitreal administration and homogenized in protein extraction buffer. After being centrifuged, the supernatant was used for protein concentration measurement and processed in sample buffer (Bio-Rad Laboratories). Equal amounts of each sample were applied to SDS-PAGE gels (Bio-Rad Laboratories), ran, and transferred to an enhanced chemiluminescent membrane. Primary antibodies used were anti-LC3 antibody (1:200; MBL, Nagoya, Japan), anti-p62 antibody (1:200, MBL), anti-p-AMPK (1:200, Thr172; Sigma-Aldrich), and anti-β-actin (1:5000; Sigma-Aldrich). Tris buffered saline with Tween 20 (T-TBS) was used as dilution solution. Secondary antibodies were diluted 1:5000 in T-TBS and they were anti-rabbit and anti-mouse antibodies (MP Biochemicals, Solo, OH, USA). An electrochemiluminescence (ECL) system was used for visualization of immunoblotting.

### Immunohistochemical Analysis

Eyeballs (from 3 rats) were enucleated 1 week after intravitreal administration, fixed, processed, and embedded in paraffin. Five micrometer transverse sections were made through the optic nerve head. Primary antibodies included anti-p-AMPK (1:100; Sigma-Aldrich), anti-Thy-1 (1:50; Santa Cruz Biotechnology, Dallas, TX, USA), and anti-neurofilament (1:100; Dako, Tokyo, Japan). The dilution was done with 1% bovine serum albumin (BSA) in PBS. Secondary antibodies were diluted in 1% BSA in PBS and they were FITC-labeled anti-rabbit and rhodamine-labeled anti-mouse antibodies (Cappel, Aurora, OH, USA). The mount medium with DAPI was put on the slide glass with the cover glass and the images were obtained by a confocal microscopy system (Zen; Carl Zeiss QEC GmbH, Köln, Germany). Captured images of p-AMPK-positive fibers in the optic nerve were then analyzed by an image software (Q-Capture Pro 7; QImaging, British Columbia, Canada). Ten different lines in one image can calculate the total image intensity. Fifty points were used to determine the background value in one image. Data from three sections of each eye were averaged for one eye, and three eyes were used for each experimental group.

### Statistical Analysis

Data are expressed as mean ± SEM. Differences among groups were analyzed by 1-way ANOVA with post hoc Tukey's Honestly Significant Difference (HSD) test. Only immunohistochemical intensity data were analyzed by the Student's *t*-test. The results were considered statistically significant when *P* < 0.05.

## Results

### Effects of Netarsudil Against TNF-Induced Axon Loss

**Figure 1.**
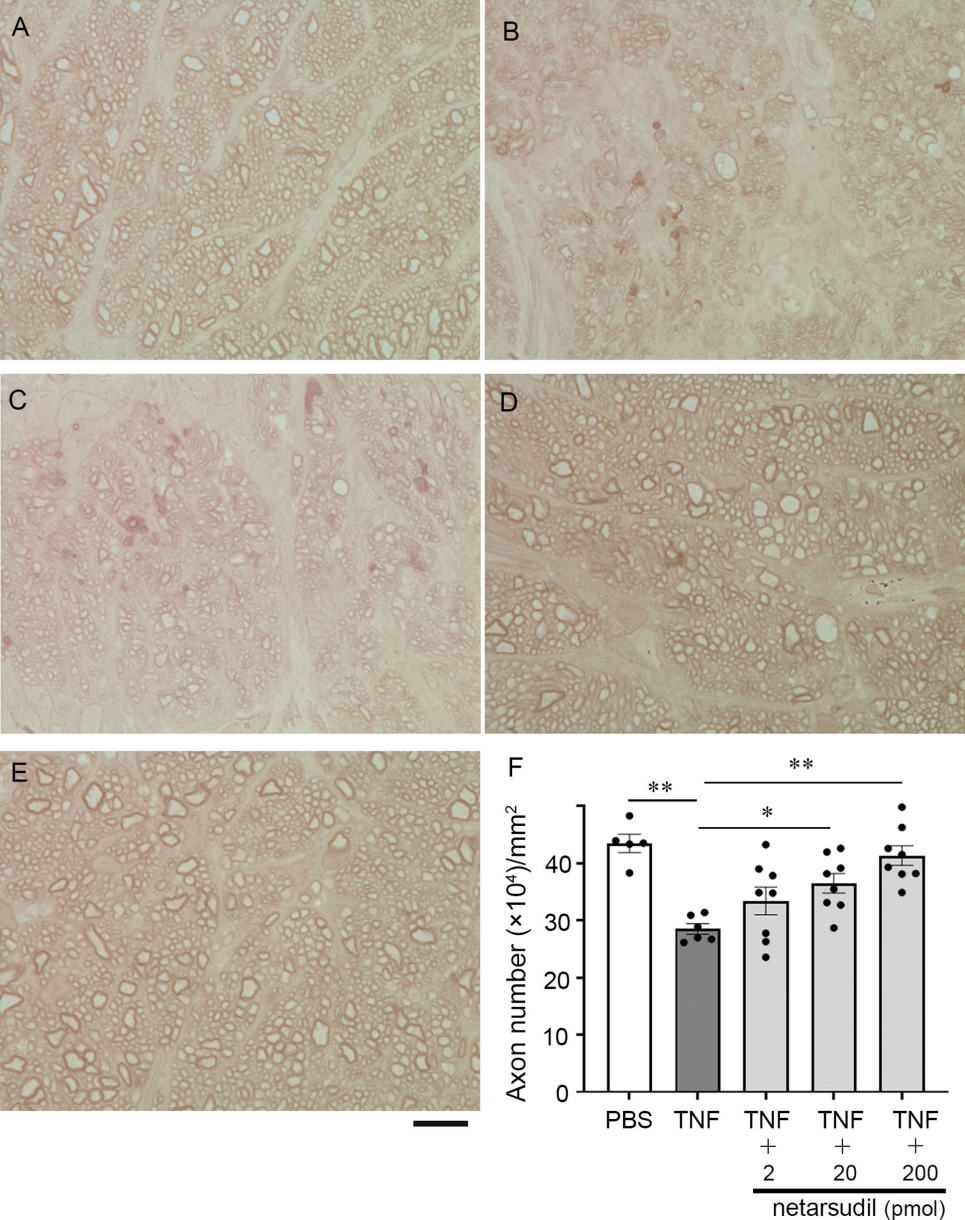
Light microscopic study of PPD-stained axons 2 weeks following intravitreal administration of (**A**) PBS, (**B**) TNF, (**C**) 2 pmol netarsudil + TNF, (**D**) 20 pmol netarsudil + TNF, or (**E**) 200 pmol netarsudil + TNF. Scale bar: 10 µm. (**F**) Quantification of axon number; *n* = 5 to 8 per experimental group. *P < 0.05, **P < 0.0005.

Consistent with our previous findings,^10^^,^^11^^,^^19^ TNF-treated group showed significant axon losses (Figs. 1B, 1F) compared with the control group (see Figs. 1A, 1F). Simultaneous administration with 2 pmol netarsudil with TNF displayed a modest tendency for protection (see Fig. 1C), although it was not significant (*P* = 0.3728 vs. TNF; see Fig. 1F). Simultaneous administration with 20 or 200 pmol netarsudil with TNF displayed apparent ameliorative effects against TNF (see Figs. 1D, 1E) and these were statistically significant (20 pmol: *P* = 0.0414 vs. TNF; 200 pmol: *P* = 0.0004 vs. TNF; see Fig 1F).

### Changes in Electron Microscopic Findings After TNF and Netarsudil Treatment

**Figure 2.**
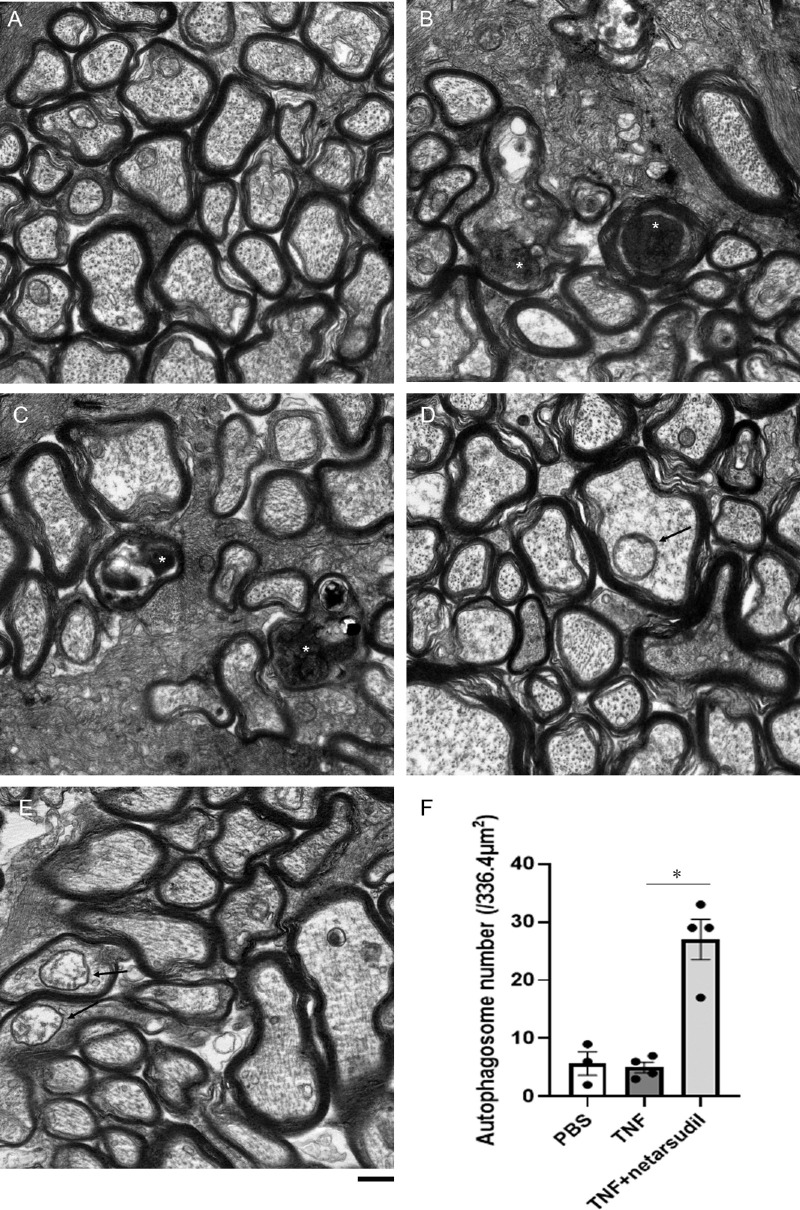
Electron microscopic study of axons 2 weeks following intravitreal administration of (**A**) PBS, (**B, C**) TNF, or (**D, E**) 20 pmol netarsudil + TNF. Degenerating axons (*white asterisks*) in the **B** and **C** TNF group. Autophagosomes inside axons (*black arrows*) in the **D** and **E** netarsudil + TNF group. Scale bar: 500 nm. (**F**) Autophagosome numbers in axons; n = 3 to 4 per experimental group. *P < 0.05.

Because we found a significant protective effect of 20 pmol netarsudil against TNF and did not find statistically significant between 20 and 200 pmol netarsudil axon numbers (*P* = 0.3092), we used this concentration to test its effect on autophagy. Normal microtubules and neurofilaments were observed inside axons in the PBS group (Fig. 2A). Degenerating axons along with the disorganization of the microtubules were seen in the TNF group (see Figs. 2B, 2C). Simultaneous administration with 20 pmol netarsudil plus TNF displayed well preserved microtubules and neurofilament structures as well as myelin structures (see Figs. 2D, 2E). Moreover, some autophagosomes were observed in this group (see Figs, 2D, 2E), and there was a significant increment of the autophagosome number compared with the TNF group (see Fig. 2F).

### Changes in LC3-II, p62, and p-AMPK Protein Levels After TNF and Netarsudil Treatment

**Figure 3.**
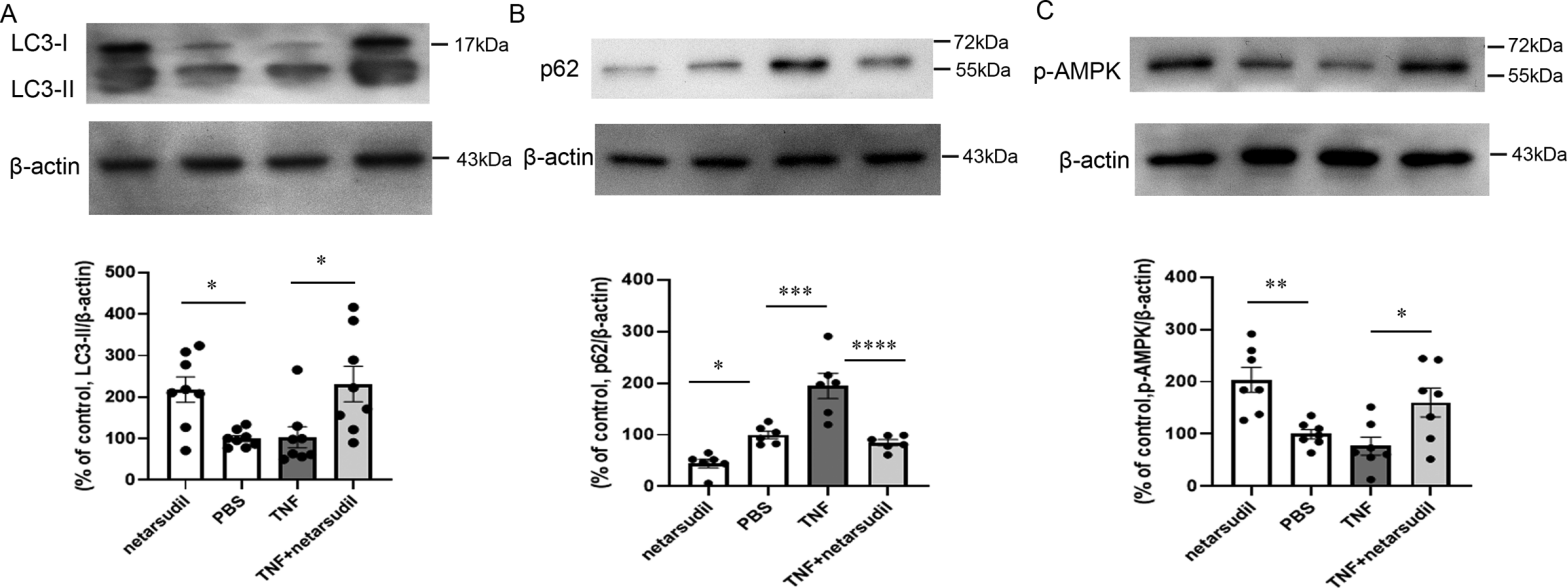
Immunoblot analysis of (**A**) LC3-II, (**B**) p62, and (**C**) p-AMPK in optic nerve samples 1 week after intravitreal administration of 20 pmol netarsudil, PBS, TNF, or 20 pmol netarsudil + TNF; *n* = 6 to 8 per experimental group. *P < 0.05, **P < 0.01, ***P < 0.001, ****P < 0.0001.

Because we previously found that axon loss started 1 week after TNF administration,^20^ the molecular events at 1 week before when axon loss becomes apparent seems to be crucial for understanding the mechanisms of axon damage. At 1 week, 20 pmol netarsudil treatment alone significantly upregulated the LC3-II level compared with the PBS group (Fig. 3A). Moreover, simultaneous administration with 20 pmol netarsudil plus TNF displayed a significant increment of the LC3-II level in the optic nerve compared with the TNF group (see Fig. 3A). In line with our previous findings,^11^ TNF administration upregulated p62 protein level in optic nerve (see Fig. 3B). This upregulation was abolished by concomitant injection of 20 pmol netarsudil plus TNF (see Fig. 3B). Furthermore, the 20 pmol netarsudil treatment alone significantly reduced the p62 level compared with the PBS group (see Fig. 3B). On the other hand, there was no significant difference in p-AMPK levels between in the TNF group and the PBS group (see Fig. 3C). In addition, 20 pmol netarsudil treatment alone significantly upregulated the p-AMPK level compared with the PBS group (see Fig. 3C). Simultaneous administration with 20 pmol netarsudil plus TNF displayed a significant upregulation of p-AMPK protein level compared with the TNF group (see Fig. 3C).

### The Effect of an AMPK Activator Against TNF-Induced Axon Loss

**Figure 4.**
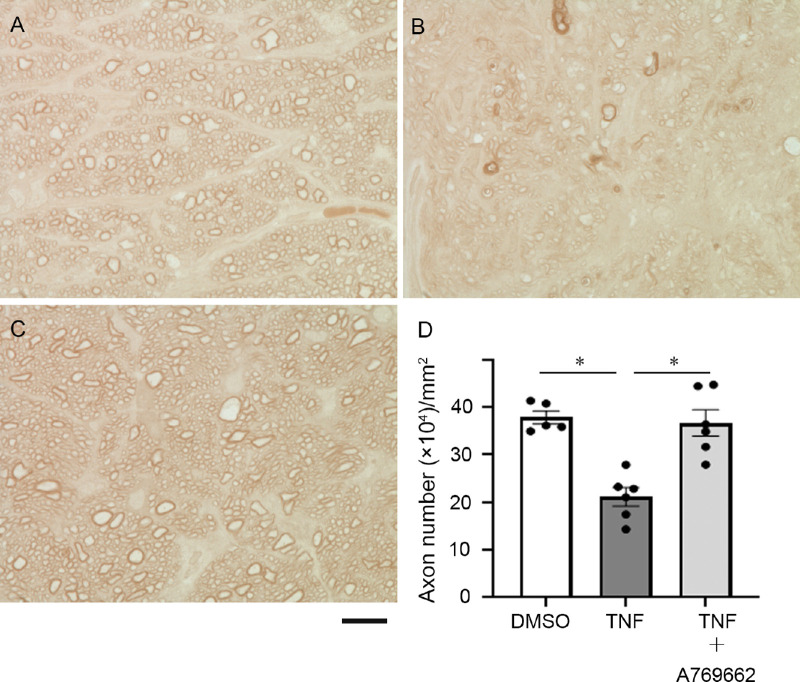
Light microscopic study of PPD-stained axons 2 weeks after intravitreal administration of (**A**) DMSO, (**B**) TNF, (**C**) 200 pmol A769662 + TNF. Scale bar: 10 µm. (**D**) Quantification of axon number; *n* = 5 to 6 per experimental group. *P < 0.0005.

A significant upregulation of p-AMPK induced by netarsudil prompted us to investigate whether an AMPK activator affects axon number in TNF-mediated optic nerve damage. Simultaneous injection with 200 pmol A769662, an activator of AMPK, plus TNF displayed substantial defensive effect against TNF-mediated axon loss (Fig. 4).

### Changes in LC3-II and p62 Protein Levels After TNF and AMPK Activator Treatment

**Figure 5.**
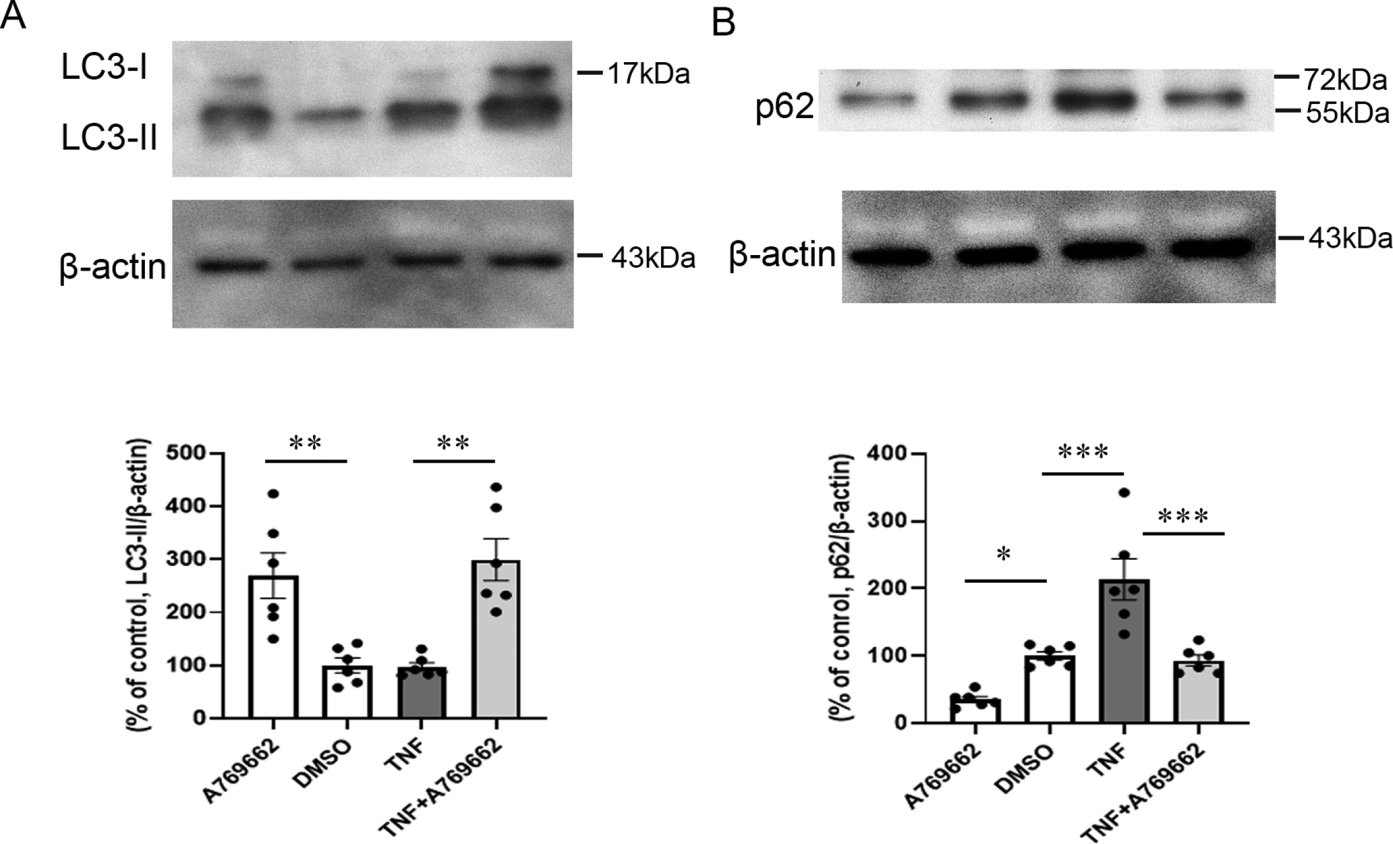
Immunoblot analysis of (**A**) LC3-II and (**B**) p62 in optic nerve samples 1 week after intravitreal administration of 200 pmol A769662, DMSO, TNF, or 200 pmol A769662 + TNF; *n* = 6 per experimental group. *P < 0.05, **P < 0.005, ***P < 0.0005.

Next, we investigated whether the AMPK activator affects autophagy in the optic nerve. The 200 pmol A769662 treatment alone significantly upregulated the LC3-II level compared with the DMSO group (Fig. 5A). Moreover, simultaneous administration with 200 pmol A769662 plus TNF displayed a significant increment of the LC3-II level compared with the TNF group (see Fig. 5A). Upregulated p62 protein level induced by TNF was significantly prevented by concomitant injection of 200 pmol A769662 plus TNF (see Fig. 5B). Furthermore, 200 pmol A769662 treatment alone significantly reduced the p62 level compared with the DMSO group (see Fig. 5B).

### Localization of p-AMPK in the Retina and Optic Nerve

**Figure 6.**
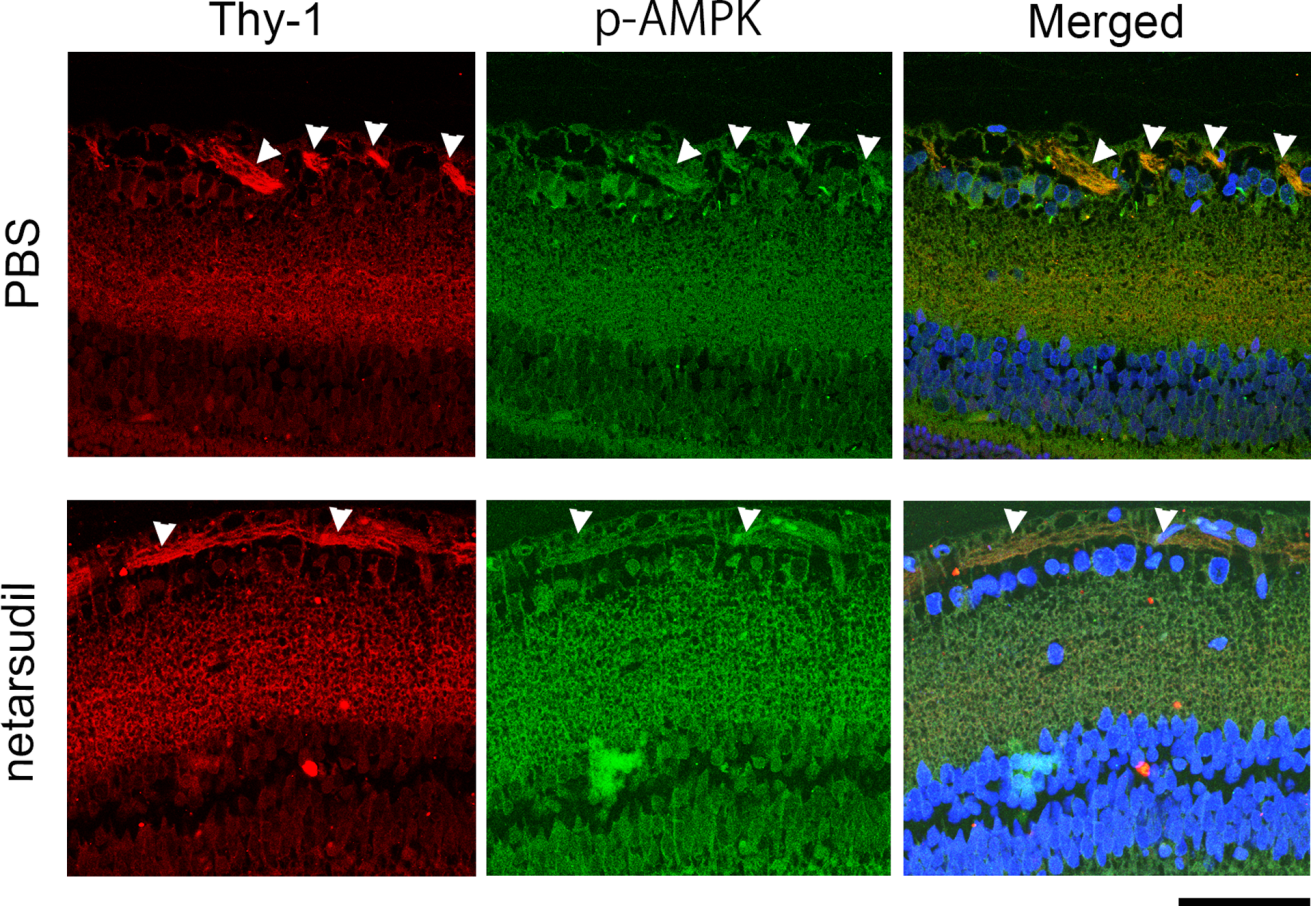
Immunohistochemical analysis of retina. p-AMPK immunoreactivity was colocalized with Thy-1-positive nerve fibers (*arrowheads*). Similar p-AMPK immunoreactive pattern was found 1 week after netarsudil treatment (*arrowheads* indicate nerve fibers). Scale bar: 50 µm.

**Figure 7.**
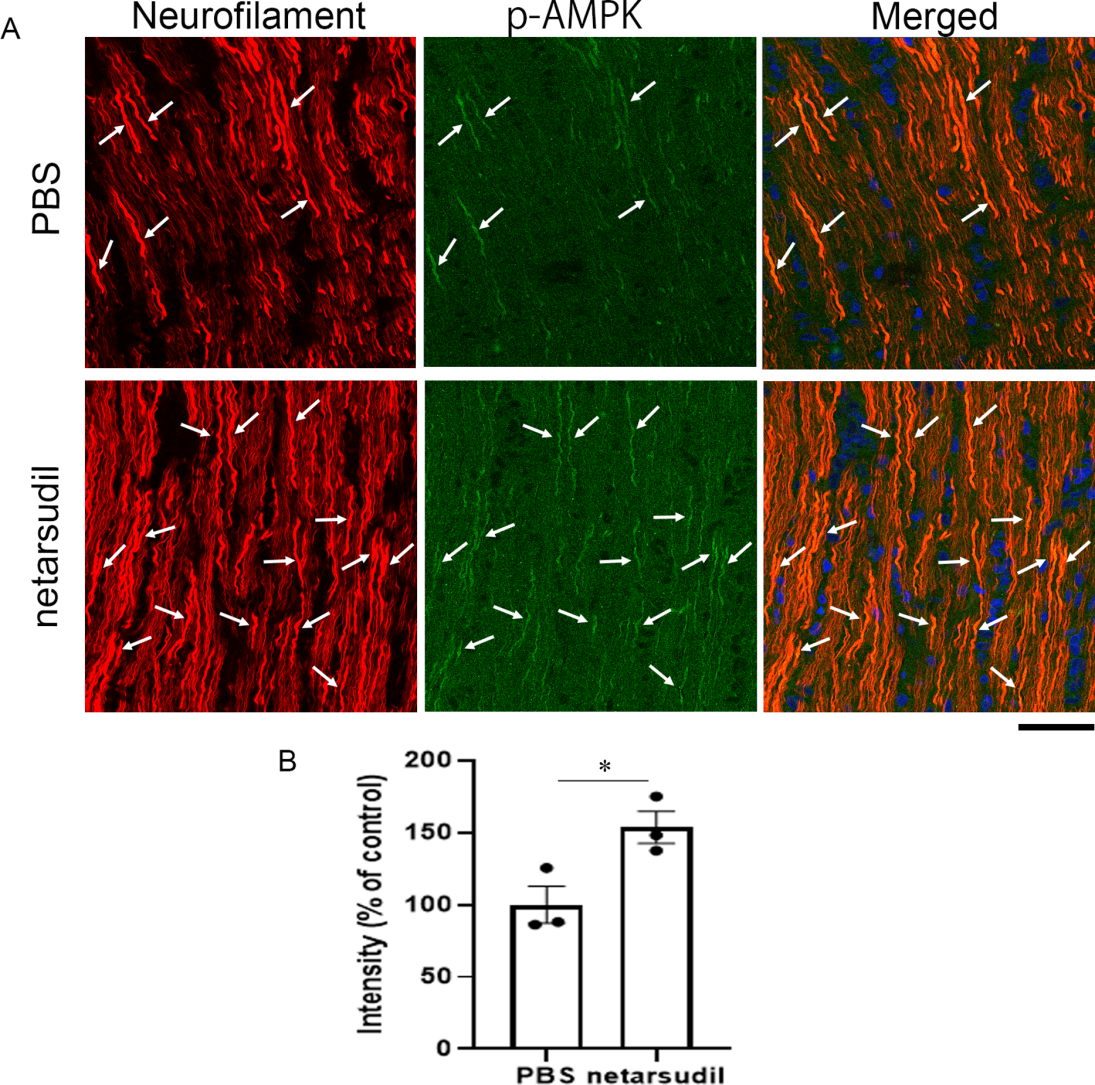
Immunohistochemical analysis of the optic nerve. (**A**) The p-AMPK immunoreactivity was colocalized with neurofilament positive fibers in PBS-treated optic nerve (*arrows*), and abundant p-AMPK immunoreactivity was found with neurofilament positive fibers 1 week after netarsudil treatment (*arrows*). Scale bar: 50 µm. (**B**) The p-AMPK signal intensity 1 week after injection of PBS or 20 pmol netarsudil; *n* = 3 per experimental group. *P < 0.05.

In the retina, the p-AMPK immunoreactivity was found in Thy-1 positive nerve fiber (Fig. 6, upper panels). A similar p-AMPK immunoreactive pattern was found after netarsudil treatment (see Fig. 6, lower panels). In the optic nerve longitudinal section, the p-AMPK immunoreactivity was found in neurofilament positive fibers (Fig. 7A, upper panels). These p-AMPK immunopositive fibers were more obvious after netarsudil treatment, and they were colocalized with neurofilament positive fibers (see Fig. 7A, lower panels). The p-AMPK immunopositive intensity was significantly increased 1 week after injection of 20 pmol netarsudil compared with the PBS group (see Fig. 7B).

### Effects of an AMPK Inhibitor on Axons in the Netarsudil Plus TNF Treatment

**Figure 8.**
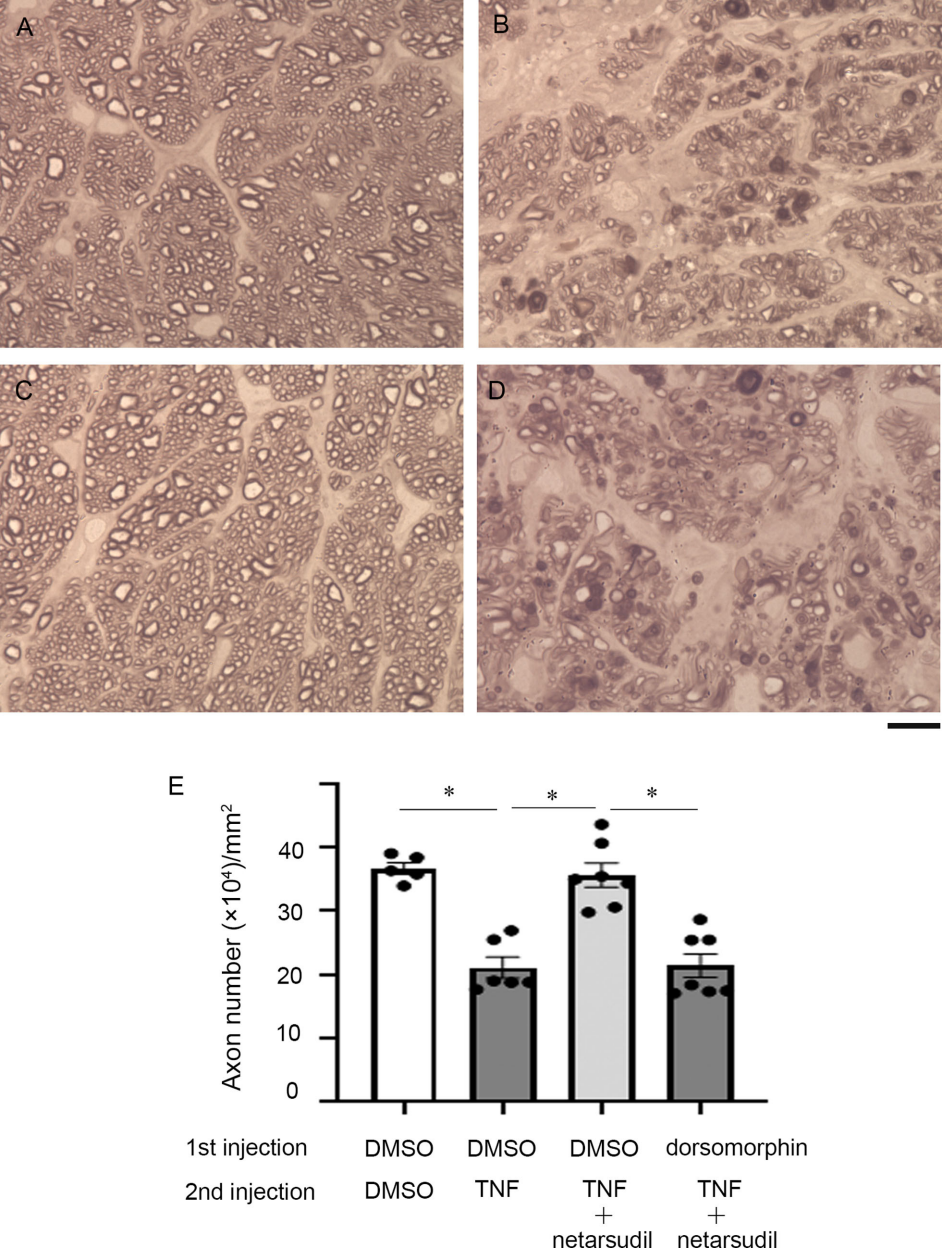
Light microscopic study of PPD-stained axons 2 weeks after the second intravitreal administration of (**A**) DMSO, (**B**) TNF, or (**C, D**) 200 pmol netarsudil + TNF. First injection of **A** to **C** DMSO or **D** 200 pmol dorsomorphin was performed 1 hour before the second injection. Scale bar: 10 µm. (**E**) Quantification of axon number; *n* = 5 to 7 per experimental group. *P < 0.0001.

To investigate whether AMPK activation is necessary for netarsudil-mediated axonal protection, pre-injection of dorsomorphin, an inhibitor of AMPK, was performed before co-injection with 200 pmol netarsudil and TNF. After the first injection of DMSO, simultaneous administration with 200 pmol netarsudil with TNF confirmed a significant protective effect (Figs. 8C, 8E) compared with the TNF group (Figs. 8B, 8E). However, pre-injection of dorsomorphin significantly suppressed netarsudil-mediated axonal protection (see Figs. 8D, 8E).

## Discussion

The present study showed a significant and dose-related protective effect of netarsudil against axon loss caused by TNF. This is in consonance with a previous study showing that topical netarsudil protected RGCs after optic nerve injury.^21^ That study found that netarsudil inhibited phosphorylation of cofilin, a downstream target of ROCK, which is localized in astrocytes, suggesting involvement of ROCK inhibition in glia, not in RGCs.^21^ We previously demonstrated that ripasudil, a ROCK inhibitor, exerted axonal protection in TNF-mediated axon damage with autophagy induction.^22^ That study confirmed the ripasudil-induced increment of autophagosome numbers inside axons, implicating the involvement of enhanced intra-axonal autophagy, not in glia. Consistent with those findings, the current study found that netarsudil increased autophagosome numbers inside axons, augmented LC3-II levels, and declined p62 levels, implicating the involvement of autophagy induction in its protective effect. LC3-I is more labile than LC3-II and the conversion of LC3-I to LC3-II is cell- and tissue-specific and dependent on the kind of autophagy induction treatment.^4^ Thus, it is proposed that levels of LC3-II should be compared to actin, but not to LC3-I.^4^ Our above findings are in congruence with past literature demonstrating that the knockdown of ROCK2 enhanced RGC survival against optic nerve axotomy and that ROCK2 shRNA augmented LC3-II levels and decreased p62 levels in primary RGC cultures.^23^ A recent review article regarding other neuronal cells, such as dopaminergic neurons, suggests that activation of ROCK appeared to stimulate disease risk factors, such as aggregation of α-synuclein, dysfunction of autophagy, and acceleration of apoptosis, and that inhibition of ROCK appeared to have potential as a treatment for neurodegenerative diseases.^24^

It is noteworthy that there is a negative regulation between ROCK1 and AMPK (i.e. when ROCK1 is inhibited, AMPK can be activated, but when ROCK1 is activated, AMPK activity is lessened in the liver).^25^ It was also shown that fasudil, a ROCK inhibitor, augmented AMPK phosphorylation in a dose-related manner in mouse myoblast cell line.^26^ The present study also revealed that netarsudil significantly augmented p-AMPK levels in the optic nerves in both the TNF-treated eyes and the PBS-treated eyes. Thus, it is possible that ROCK inhibition leads to AMPK activation in not only non-neuronal cells but also neurons. Moreover, it is interesting to note that resveratrol exerted ameliorated effects against cerebral ischemia through stimulation of AMPK-autophagy signaling.^27^ Furthermore, it was demonstrated that naringenin, a principal flavonoid, rescued neuronal cells against amyloid-β through AMPK activation-mediated upregulation of autophagy.^28^ Thus, some reports implied that AMPK activation and autophagy upregulation are beneficial pathways in preventing neuronal damages. In addition, there have been some studies showing protective effects of AMPK activators in neurons. For example, treatment with A769662, the AMPK activator, significantly improved hyperglycemia induced neuronal injury in vivo and substantially enhanced neurite outgrowth of N2A cells.^29^ That study also showed that A769662 augmented LC3-II levels in high glucose-insulted N2A cells.^29^ In the present study, A769662 augmented LC3-II levels and declined p62 levels in the optic nerve. It was shown that the treatment of AICAR, the AMKP activator, ameliorated depressive behavior and promoted hippocampal neurogenesis in the olfactory bulbectomized mice.^30^ In the current study, we observed a significant protective effect of A769662 against axon loss induced by TNF. Therefore, it is plausible that activation of AMPK has a beneficial effect on certain types of neurodegenerations.

In the eyes, p-AMPK has been found in the neural retina, including photoreceptor cells^31^ and RGCs.^32^ Our current study also found that p-AMPK exists in RGC axons in the retina and optic nerve and that these axonal immunoreactivities were increased by netarsudil. Although one possibility is that the protective effect of netarsudil may be directly due to ROCK inhibition, the current study demonstrated that netarsudil-mediated axonal protection was significantly suppressed by dorsomorphin, the AMPK inhibitor. A previous protein kinase assay study showed that 1 µM dorsomorphin/compound C inhibits 73% AMPK activity and that this dose results in 27% activity remaining.^33^ The dose of dorsomorphin in the current study is 2 µL of 100 µM and presumable the vitreous concentration is about 10 µM. However, caution will be needed for the interpretation because dorsomorphin can inhibit some other kinases not only AMPK.^34^ Nonetheless, taken together, these findings propose that axonal protection by netarsudil may be associated with AMPK activation and autophagy induction in TNF-induced optic nerve degeneration.
